# Taurine/Pilocarpine Interaction in the Malnourished Rat Brain: A Behavioral, Electrophysiological, and Immunohistochemical Analysis

**DOI:** 10.3389/fnins.2019.00981

**Published:** 2019-09-18

**Authors:** Elian da Silva Francisco, Rosângela Figueiredo Mendes-da-Silva, Cássia Borges Lima de Castro, Geórgia de Sousa Ferreira Soares, Rubem Carlos Araújo Guedes

**Affiliations:** ^1^Departamento de Nutrição, Universidade Federal de Pernambuco, Recife, Brazil; ^2^Departamento de Educação Física, Centro Universitário Católico de Quixadá, Quixadá, Brazil

**Keywords:** taurine, pilocarpine, anxiety-like behavior, blood glucose, brain excitability, glial cells, nutritional deficiency, spreading depression

## Abstract

This study aimed to evaluate the possible protective role of taurine on anxiety-like behavior, brain electrical activity and glial cell immunoreactivity in well-nourished and malnourished rats that were treated with a subconvulsing dose of pilocarpine. Newborn Wistar rats were subjected to normal or unfavorable lactation conditions, represented by the suckling of litters with 9 or 15 pups, resulting in well-nourished and malnourished animals, respectively. Each nutritional group was split into five subgroups that were treated from postnatal day (PND) 35 to 55 with 300 mg/kg/day of taurine + 45 mg/kg/day of pilocarpine (group T + P), taurine only (group T), pilocarpine only (group P), vehicle control (group V), or not treated control (group naïve; Nv). At PND56-58, the groups were subjected to the elevated plus-maze behavioral tests. Glycemia was measured on PND59. Between PND60 and PND65, the cortical spreading depression (CSD) was recorded in the cerebral cortex, and the levels of malondialdehyde and microglial and astrocyte immunoreactivity were evaluated in the cortex and hippocampus. Our data indicate that treatment with taurine and pilocarpine resulted in anxiolytic-like and anxiogenic behavior, respectively, and that nutritional deficiency modulated these effects. Both treatments decelerated CSD propagation and modulated GFAP- and Iba1-containing glial cells. Pilocarpine reduced body weight and glycemia, and administration of taurine was not able to attenuate the effects of pilocarpine. The molecular mechanisms underlying taurine action on behavioral and electrophysiological parameters in the normal and altered brain remain to be further explored.

## Introduction

Taurine is an amino sulfonic acid that is found abundantly in several areas of the mammalian central nervous system. It is a structural analog of the inhibitory transmitter gamma-aminobutyric acid (GABA), which is recognized as one of the most important inhibitory amino acids distributed in the brain tissue (Barbeau et al., 1975; Yakimova et al., 1996; Ripps and Shen, 2012). Taurine mediates a myriad of physiological processes in the nervous system, including neuromodulation, maintenance of calcium homeostasis, and neuronal proliferation and differentiation (Chen et al., 1998). Administration of exogenous taurine improves glucose homeostasis in genetically obese animals (Santos-Silva et al., 2015), promotes an anxiolytic-like behavioral profile (Murakami and Furuse, 2010; Francisco and Guedes, 2015), acts as an antioxidant and anti-inflammatory molecule (Oliveira et al., 2010; Marcinkiewicz and Kontny, 2014) and rescues hippocampal long-term potentiation (LTP) from ammonia-induced impairment (Chepkova et al., 2006). Furthermore, taurine reportedly acts as a neuroprotectant in epilepsy, reducing or abolishing seizures (Junyent et al., 2011; Oja and Saransaari, 2013; see a recent review in Hrnčić et al., 2018).

The acute systemic injection of pilocarpine in rodents constitutes an effective, experimental model largely used to study the pathophysiology of seizures and to identify potential therapeutic agents for the treatment of epilepsy (Santos et al., 2000). This model was first described by Turski et al. (1983a, b)); it consists of the single administration of a high dose (300–380 mg/kg; Guedes and Cavalheiro, 1997) or various consecutive low doses of pilocarpine until induction of *status epilepticus* (Glien et al., 2001). This acute phase is followed by a condition of permanent recurrent spontaneous seizures, altering the central nervous system structure and function (Turski et al., 1989) with behavioral and electroencephalographic changes that are similar to those observed in human temporal lobe epilepsy. In the last decade, some studies had given special attention to possible effects of subconvulsing doses of pilocarpine when administered acutely. Under this condition, no behavioral or electrocorticographic changes indicative of seizures were observed (Guedes and Vasconcelos, 2008). However, under subconvulsing paradigms several reports have described anxiety-like behavioral profiles (Duarte et al., 2014; Francisco and Guedes, 2018), reductions in glycemia (Francisco and Guedes, 2018), increases in brain oxidative stress (Mendes-da-Silva et al., 2018) and impairment of propagation of the excitability-related phenomenon known as cortical spreading depression (CSD) along the cortical rodent tissue (Francisco and Guedes, 2018; Mendes-da-Silva et al., 2018).

Cortical spreading depression is a brain phenomenon that is based on neuronal and glial depolarization and is influenced by conditions that modify neural excitability, including cholinergic agonists (Guedes and Cavalheiro, 1997). CSD has been related to excitability-associated diseases such as migraine with aura (Lauritzen, 1994); CSD in the injured human brain was first shown by Mayevsky et al. (1998) and Strong et al. (2002). CSD has been also associated with ischemic stroke (Dohmen et al., 2008), traumatic brain injury (Hartings et al., 2009), subarachnoid hemorrhage (Dreier, 2011), multiple sclerosis (Pusic et al., 2015), and epilepsy (Vinogradova et al., 2006; Dreier et al., 2012). Evidence suggests that chemicals such as potassium (Grafstein, 1956) or glutamate (Van Harreveld, 1959; Marrannes et al., 1988; Pietrobon and Moskowitz, 2014; Hertelendy et al., 2018), as well as various other neurotransmitters and neuromodulators (Ayata and Lauritzen, 2015; Guedes et al., 2017) might be involved in CSD, either eliciting, or modulating the phenomenon. Experimental evidence demonstrated that CSD can potentiate the brain’s spontaneous and evoked electrical activity, both *in vitro* (Footitt and Newberry, 1998) and *in vivo* (Guedes et al., 2005; Souza et al., 2015). Under conditions of environmental, nutritional and pharmacological manipulations, our group has extensively employed the CSD model to evaluate the proper functioning of the brain in health and disease. When occurring early in life, such conditions can affect brain development and functioning, and can substantially alter the ability of the brain to produce and propagate CSD (see Guedes, 2011 for an overview). Recently, we demonstrated that the chronic administration (21 days) of a very low, subconvulsive dose of pilocarpine (45 mg/kg/day) is able to counteract CSD, and this effect is modulated by nutritional deficiency (Francisco and Guedes, 2018).

In the present study we tested the hypothesis that taurine modulates the CSD effects of pilocarpine, in association, or not, with early malnutrition. In addition, we investigated the taurine/pilocarpine/malnutrition interaction on anxiety-like behavior, fasting glycemia and oxidative stress. Finally, some structural correlates of this interaction were investigated by correlating the experimental treatments with the astrocytic and microglial immunostaining pattern in the cerebral cortex and hippocampus.

## Materials and Methods

### Animals

All experimental procedures were previously approved by the Institutional Ethics Committee for Animal Research of the Federal University of Pernambuco (approval protocol no. 23076.015655/2015-99), whose norms comply with the norms established by the National Institutes of Health Guide for Care and Use of Laboratory Animals (Bethesda, MD, United States). Newborn Wistar rats of both sexes, born from different dams, were assigned to be suckled under normal or unfavorable lactation conditions, represented respectively by litters with nine pups (L_9_ groups) and litters with 15 pups (L_15_ groups), as previously described (Francisco and Guedes, 2015). Weaning occurred on postnatal day (PND) 21, when pups were separated by sex and housed in polypropylene cages (51 cm × 35.5 cm × 18.5 cm; three rats per cage) under a 12-h light:12-h dark cycle (lights on at 6:00 a.m.), controlled temperature (23 ± 1°C), and with free access to water and the same commercial lab chow, with 23% protein, that was offered to their dams during the lactation period (Purina, Ltd.). In this study, we analyzed data from male pups only, i.e., 46 L_9_ and 45 L_15_ rats.

### Administration of Pilocarpine and/or Taurine

Pilocarpine hydrochloride, scopolamine methyl nitrate, and taurine were purchased from Sigma-Aldrich (St. Louis, MO, United States). All solutions were prepared daily, shortly before the injections, dissolved in 0.9% saline and administered from PND35 to PND55. Each lactation condition gave rise to the following five subgroups: (1) Naïve (control group, without any treatment; *n* = 10 L_9_ and 7 L_15_ rats); (2) Vehicle (second control group; treated with saline via i.p. and gavage; *n* = 10 L_9_ and 8 L_15_ rats); (3) Taurine (300 mg/kg/day via gavage; *n* = 9 L_9_ and 9 L_15_ rats), as previously described (Francisco and Guedes, 2015); (4) Pilocarpine (45 mg/kg/day via i.p.; *n* = 9 L_9_ and 10 L_15_ rats), following Mendes-da-Silva et al. (2018); (5) Taurine plus pilocarpine (300 mg/kg/day via gavage and 45 mg/kg/day via i.p, respectively; *n* = 8 L_9_ and 11 L_15_ rats). Scopolamine methyl nitrate, a muscarinic receptor antagonist, was administered (1 mg/kg/day via i.p.) in all groups, with the exception of the naïve group, 30 min before pilocarpine or saline administration to prevent the peripheral cholinergic effects elicited by pilocarpine (Peixinho-Pena et al., 2012). Immediately following pilocarpine administration, the animals were observed over 1 h to confirm the absence of seizures, as evaluated by the Racine scale (Racine, 1972). At this low dose of pilocarpine, 45 mg/kg/d, no behavioral signs of epilepsy were observed in the animals (all rats presented with Racine’s score of zero).

### Body and Brain Weights

Body weight was measured using a Filizola MF-3/1 electronic scale (3.0 kg capacity and precision of 0.5 g) at PND7, PND21, PND35, PND49, and PND60. The brain weights were obtained using an analytical balance (Shimadzu, model AUY220, with a sensitivity of up to 0.1 mg) at the end of the transcardiac perfusion procedure (see item 2.8).

### Elevated Plus-Maze Test

The elevated plus-maze test (EPM) was conducted at PND56-58. The cross-shaped EPM apparatus was made of varnished wood and consisted of four arms (49 cm × 10 cm each) elevated 55 cm above the ground. Two of the arms were opened and the other two arms were closed (lateral walls of 50 cm of height), arranged perpendicular to the open ones. The arms of the apparatus were joined by a central 10 cm × 10 cm square platform. At the beginning of the test, each animal was placed individually in the central area of the labyrinth, with the head directed toward one of the open arms. Each animal was allowed to freely explore the labyrinth for 5 min, under dim light and in a sound-attenuated room. Before each test, the EPM apparatus was wiped with a paper cloth soaked in 70:30 ethanol:water solution. The animal’s behavioral activity was recorded by a video camera. The video-recorded activity was stored in a computer and subsequently analyzed with the aid of the software ANY maze^TM^ (version 4.99 m), as previously described (Lima et al., 2017). The following parameters were considered: number of expelled fecal boluses, total distance traveled, total immobility time, number of entries into the open arms and the time spent in the open arms.

### Analysis of Blood Glucose

As reported formerly (Francisco and Guedes, 2015), on PND59 the animals were fasted for 6 h and a drop of blood was collected from the animal’s tail and used for measuring the blood glucose level using a portable glucose meter (G-TECH free).

### CSD Recording

On the day of the electrophysiological recording (PND60–PND65), animals were anesthetized with a mixture of 1000 mg/kg urethane plus 40 mg/kg chloralose injected intraperitoneally. The level of anesthesia was monitored as previously described (Souza et al., 2015). Three trephine holes were drilled on the right side of the skull, aligned in the frontal-to-occipital direction and parallel to the midline. One hole was positioned on the frontal bone (2 mm in diameter) and used to apply the stimulus (KCl) to elicit CSD. The other two holes were positioned on the parietal bone (3–4 mm in diameter) and used to record the propagating CSD wave. Rectal temperature was continuously monitored and maintained at 37 ± 1°C by means of a heating blanket. The electrophysiological recording session lasted 6 h. We used two Ag–AgCl agar–Ringer electrodes (one in each hole) against a common reference electrode of the same type, placed on the nasal bones. The two initial recording hours constituted the baseline period, during which no KCl stimulus was applied and, consequently, no CSD was elicited. In the remaining four recording hours, CSD episodes were elicited at 30-min intervals by a 1-min application of a cotton ball (1–2 mm in diameter) soaked with 2% KCl solution (approximately 270 mM) to the anterior hole drilled at the frontal region. The ECoG and the DC (direct current) slow potential variation that is typical of CSD were continuously recorded on the cortical surface (on the intact dura mater) through a digital recording system (Biopac MP 150, Goleta, CA, United States). For each animal, the amplifier’s gain was kept constant over the entire recording session, as previously reported (Lopes-de-Morais et al., 2014).

We calculated the CSD velocity of propagation from the time required for a CSD wave to pass the distance between the two cortical electrodes. In the two recording locations, we used the initial point of each DC-negative rising phase as the reference point to calculate the CSD velocities. In addition, we calculated the amplitude and duration of the CSD waves, as previously reported (Lima et al., 2017). As the basis for assessing the occurrence of CSD-dependent potentiation of spontaneous electrical activity, in each animal we compared the amplitudes of the ECoG before (baseline recording) and after starting to regularly elicit CSD. For this comparison, we analyzed six 10-min samples of the record at six time points of the ECoG, i.e., two samples from the baseline period and four samples from the CSD period. These samples were analyzed offline with the aid of an algorithm implemented in MATLAB^TM^ software (The Mathworks, Natick, MA, United States), version R2011B. This algorithm calculates the average amplitude of the ECoG waves. For each animal, the averaged ECoG amplitude was normalized in relation to the lowest sample value, which was considered equal to 1, expressed in relative units and compared before and after the episodes of CSD, as a basis for analyzing the occurrence of potentiation of the spontaneous electrical activity, as reported by our group previously (Lopes-de-Morais et al., 2014; Souza et al., 2015).

### Lipid Peroxidation Analysis

After the CSD recording session, 50 of the still-anesthetized animals (26 L_9_ and 24 L_15_ rats) were decapitated; their brains were rapidly removed and frozen. The cortical tissue was homogenized in a cold Tris buffer solution and centrifuged for 10 min at 1000 *g* at 4°C. Supernatants were used to estimate the lipid peroxidation by measuring malondialdehyde (MDA) levels using a thiobarbituric acid-reactive substances-based method (Ohkawa et al., 1979), which is a parameter to evaluate the lipid peroxidation. The reaction was developed by the sequential addition of 40 μl 8.1% sodium dodecyl sulfate, 300 μl 20% acetic acid (pH 3.5), and 300 μl 0.8% thiobarbituric acid solutions to the 300 μl homogenate aliquot in a boiling water bath for 50 min. After cooling the tubes with tap water, 300 μl of n-butanol was added to the sample. The tubes were centrifuged at 2500 *g* for 10 min, and the organic phase was read at 532 nm using a plate reader. Measurements were carried out in triplicate. Total protein concentrations were determined based on the Bradford protein assay; bovine serum albumin was used as a standard. MDA concentrations were determined by using 1,1,3,3-tetraethoxypropane as the standard and expressed as μg/mg protein, as previously reported (Mendes-da-Silva et al., 2014). All measurements were performed in triplicate.

### Immunohistochemistry

After the CSD recording session, forty-one of the still-anesthetized animals were perfused with 0.9% saline solution followed by 4% paraformaldehyde diluted in 0.1M phosphate-buffered saline (pH 7.4). After being immersed in the fixative for 4 h, the brains were subjected to cryoprotection in sucrose solutions of increasing concentrations of 10%, 20% and 30%. Longitudinal serial sections (40-μm thickness) from the left (CSD-free) hemisphere were obtained at −20°C using a cryoslicer (Leica, 1850). One of the 41 animals was processed as a negative control for the immunolabeling. The remaining 40 rats included 20 from the L_9_ and 20 from the L_15_ condition. In each lactation condition, 4 rats were from each one of the five treatment groups. Their brains were processed for microglia immunolabeling with anti- ionized calcium binding adapter molecule 1 (Iba1) antibody and astrocyte immunolabeling with anti-GFAP antibody.

For microglia immunostaining, sections were immunolabeled with a polyclonal antibody against Iba-1 (1:1500; anti-Iba-1, #019-19741; Wako Pure Chemical Industries, Ltd., Osaka, Japan). Free-floating sections were subjected to endogenous peroxidase blocking (2% H_2_O_2_ in 70% methanol for 10 min) and the sections were incubated for 1 h in blocking buffer (BB) solution containing 0.05M Tris-buffered saline (TBS; pH 7.4), 10% fetal calf serum, 3% bovine serum albumin, and 1% Triton X-100. The sections were then incubated overnight at 4°C with rabbit anti-Iba-1 (1:1500 diluted in BB solution). After three washes with TBS + 1% Triton X-100, sections were incubated at room temperature for 1 h with biotinylated anti-rabbit (1:500) secondary antibodies. Sections were then rinsed in TBS + 1% Triton X-100 and incubated with horseradish peroxidase streptavidin (1:500). The peroxidase reaction was visualized by incubating the sections in Tris buffer containing 0.5 mg/ml 3,3′-diaminobenzidine) and 0.33 μl/ml H_2_O_2_. The experimental protocol used for astrocyte immunostaining was similar to that applied for microglial labeling, mentioned above, with the following change: the primary antibody that was used (polyclonal rabbit anti-GFAP-D1F4Q-XP RABBIT MAB; Dako, Denmark) was specific for astrocyte labeling at the ratio of 1:2400.

**FIGURE 1:**
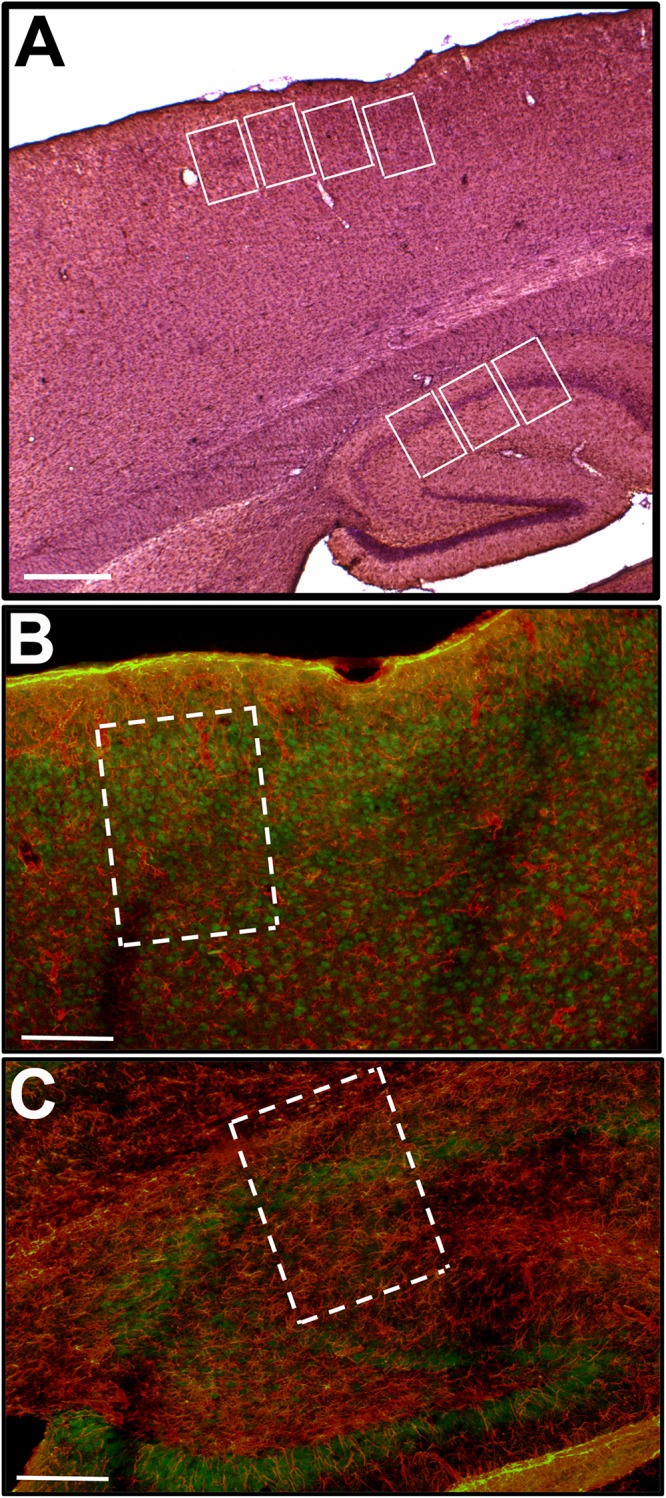
**(A)** Shows a low magnification (2.5×) image of a representative parasagittal section immunostained for GFAP and counterstained with 5% Hematoxylin. The white rectangles illustrate the position of the sampling windows (470 μm × 340 μm) in the cerebral cortex and CA1 region of hippocampus. Double staining for GFAP (red) and Neu-N (green) was performed in order to better define the cytoarchitectonic limits of layers 2 and 3 in the cerebral cortex (**B**; 10×) as well as the four layers (Stratum Oriens, Stratum Pyramidalis, Stratum Radiatum, and Stratum Lacunosum Moleculare) in the hippocampus (**C**; 10×), where the quantitative analysis was carried out. Scale bars equal 500 μm in **(A)**, and 200 μm in **(B,C)**.

Finally, the sections were mounted, dehydrated in graded alcohols, and coverslipped with Entellan^®^ after xylene treatment. Densitometric analysis was performed on four parallel longitudinal sections for each animal. A Leica DMLS microscope coupled to a Samsung high-level color camera (model SHC-410NAD) was used to obtain digital images from the brain sections. Images from selected regions of interest (Figure 1) of the parietal cortex and CA1 hippocampus stained for Iba1 and GFAP were obtained using a 20 × microscope objective. In Figure 1, the protocol for double labeling was as follows: the sections were incubated simultaneously with mouse monoclonal anti-GFAP (Sigma-Aldrich; United States, #G3893, 1:1000) and rabbit polyclonal anti-neuN (Novus Biologicals, United States, #NBP1-77686, 1:200) for 18 h. Then, they were rinsed in phosphate buffer 0.1M (PB), pH 7.4 followed by incubation for 4 h with Cy-3 conjugated 546 labeled anti-mouse IgG and FITC-conjugated 488 anti-rabbit IgG (1:500; Jackson ImmunoResearch Labs, United States). After washing twice in PB, they were mounted onto gelatin coated slides and dried at 50°C for 5 min, cleared in xylene for 1 min and coverslipped with Entellan (Merck-Millipore, United States). Digital images were obtained using an epifluorescence microscope (Nikon coupled to a high-level color camera Model SHC-410NAD). The CA1 area and the parietal neocortex were chosen based on morphological and pharmacological studies in pilocarpine-treated animals (Curia et al., 2008; Rossi et al., 2013; Arisi et al., 2015; García-García et al., 2017). In each section, photomicrographs of four fields within the parietal cortex (layers 2 and 3) and three fields of the CA1 hippocampal region (including Stratum Oriens, Stratum Pyramidalis, Stratum Radiatum, and Stratum Lacunosum Moleculare) were analyzed, using the ImageJ software (National Institutes of Health, United States, version 1.46r). Care was taken to obtain the digital images using the same light intensity. The color images were first converted into a gray scale. Based on the color difference, an algorithm of the program, devoted to area selection, identified the darker areas (marked cells) in relation to the lighter areas (background), and the total marked area was calculated. The threshold for selection was manually adjusted such that the background was not marked. All sections that were photographed at the same magnification displayed the same total area in the photographs; therefore, the ratios between the labeled cells’ area and the total picture area could be directly compared. The labeled area was expressed as percentage of the total area in the picture. The immunoreactivity intensity was obtained in the program by calculating the mean gray value (MGV) within the selected area. The MGV can vary numerically from 0 (darkest) to 255 (lightest). Therefore, the reactivity intensity was given by the difference (255-MGV). By multiplying this value by the marked area, we came to the figure (arbitrary unit) that indicated how much of the gray area in the image was due to cell labeling, i.e., mathematically, the more intense the labeling, the greater is the arbitrary unit value. Total immunoreactivity expressed as arbitrary units as well as the percentage of the area occupied by the immunolabeled cells were analyzed, as previously reported (Lima et al., 2017).

### Statistical Analysis

Results in all groups are expressed as the means ± standard deviations (SD). Intergroup differences and interactions were first analyzed using a two-way ANOVA and thereafter a MANOVA. In the ANOVA analysis, we consider, as factors, nutritional status (L9 and L15) and treatment (naïve, vehicle, taurine, pilocarpine, and taurine + pilocarpine). In the MANOVA analysis we included nutritional status (L_9_ and L_15_), pilocarpine administration and taurine treatment as factors. This was followed by a *post hoc* test (Holm–Sidak) where indicated. ECoG amplitude values before and after CSD for each animal were normalized and expressed in relative units. Differences in these amplitudes, before and after CSD, were analyzed with the paired *t*-test, using ANOVA followed by the Holm–Sidak test for intergroup comparisons when indicated. Differences with *p* < 0.05 were accepted as significant. Since MANOVA confirmed the differences that had been previously indicated by two-way ANOVA, we kept the ANOVA statistics figures in the description of results.

## Results

The two control groups – naïve (no treatment) and vehicle (that received gavage and intraperitoneal injection of saline and scopolamine) presented comparable values.

### Body and Brain Weights

**FIGURE 2:**
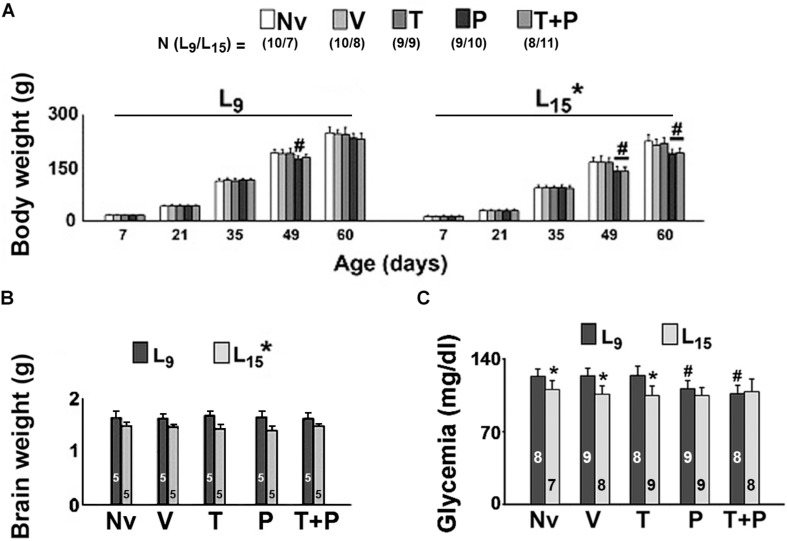
Body **(A)** and brain weights **(B)**, and glycemia **(C)** of male rats previously suckled in litters with 9 and 15 pups (respectively, L_9_ and L_15_ condition). Naïve (Nv) = no treatment; Vehicle (V) = scopolamine methyl nitrate 1 mg/kg/day + saline; Taurine (T) = 1 mg/kg/day scopolamine methyl nitrate + 300 mg/kg/day of taurine; Pilocarpine (P) = 1 mg/kg/day scopolamine methyl nitrate + 45 mg/kg/day of pilocarpine; Taurine + pilocarpine (T + P) = 1 mg/kg/day scopolamine methyl nitrate + 300 mg/kg/day of taurine + 45 mg/kg/day of pilocarpine. All drugs were dissolved in 0.9% saline. Administration occurred from postnatal days 35 to 55. Data are mean ± standard deviation. ^∗^*p* < 0.001 compared with the corresponding L_9_ condition. ^#^*p* < 0.001 compared with the control groups in the same lactation condition (ANOVA plus Holm–Sidak test). In panels **(B,C)**, the numbers in each bar indicate the sample size of each measurement.

As shown in Figure 2A, in all treatment groups (Nv, V, T, P, and T + P) ANOVA showed a main effect of the lactation condition on body weight (*p* < 0.001). The L_15_ animals presented with lower body weights compared with the corresponding L_9_ groups. In the control condition (naïve and vehicle groups) the weight reduction ranged from 11.9 to 32.5%. In the L_9_ condition, intergroup differences were observed at PND49 only [*F*(4,85) = 15.048; *p* < 0.001]. At that age, the treatment with pilocarpine was associated with a weight reduction compared to the respective L_9_ control groups. In the unfavorable (L_15_) lactation condition, pilocarpine and taurine + pilocarpine treatments reduced body weight at PND49 and PND60 [*F*(4,79) = 11.993; *p* < 0.001].

For the brain weight data (Figure 2B), ANOVA showed a main effect of the lactation condition on brain weight [*F*(1,41) = 51.659, *p* < 0.001]. L_15_ animals presented with a lower brain weight compared to the respective L_9_ groups. The average weight reduction was 17.03% and was independent of the treatment.

### Blood Glucose Level

In the L_15_ condition, the Nv, V, and T groups displayed significantly lower glycemia than the corresponding L_9_ groups [*F*(1,77) = 33.484; *p* < 0.001]. Treatment with P and T + P reduced blood glucose levels in the L_9_, but not in the L_15_ groups [*F*(4,77) = 4.122; *p* = 0.004] compared with the corresponding Nv, V, and T groups. Data on glycemia are illustrated in Figure 2C.

### Behavioral Activity in the Elevated Plus-Maze

**FIGURE 3:**
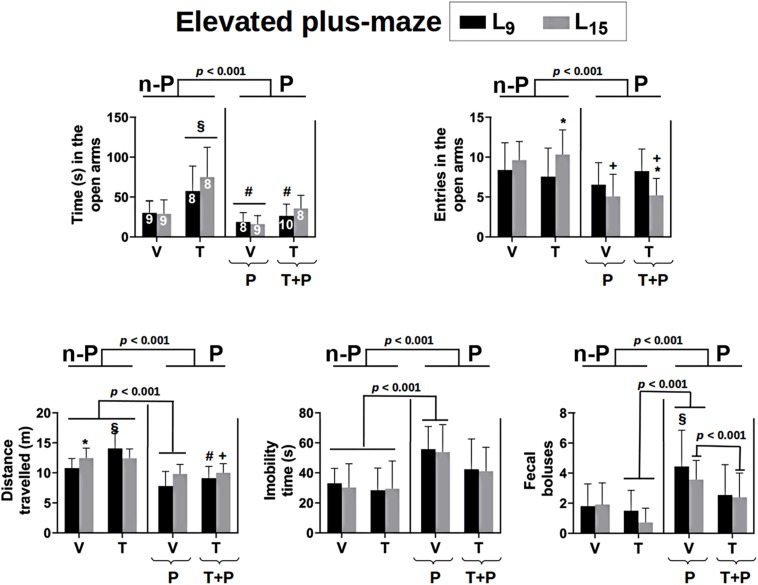
Behavioral activity in the elevated plus-maze test of 56-day-old male rats previously suckled in litters with 9 and 15 pups (respectively, L_9_ and L_15_ condition). Group description is as in Figure 2. n-P = no pilocarpine; P = pilocarpine; T = taurine; V = vehicle. Data are mean ± standard deviation. ^∗^*p* < 0.001 compared to the corresponding L_9_ condition. ^§^ *p* < 0.001 in comparison to groups Nv and V in the same lactation condition. ^#^*p* < 0.001 compared to group T of the same lactation condition. ^+^*p* < 0.001 compared to groups V and T of the same lactation condition (ANOVA plus Holm–Sidak test). The number in each bar of the upper-left graphic indicates the sample size, which applies to all graphics.

The effect of administration of taurine and/or pilocarpine on behavioral activity in the EPM test is shown in Figure 3. Regarding the time spent in the open arms, ANOVA indicated a main effect of treatment [*F*(4,76) = 10.653, *p* < 0.001]. In the L_9_ condition, the Holm–Sidak test revealed that the group treated with taurine remained longer in the open arms compared to the other groups in the same lactation condition. In the L_15_ condition, the taurine-treated rats remained significantly longer in the EPM open arms, compared to the Nv, V and P groups, but not to the T + P groups.

Regarding the number of entries into the open arms, ANOVA revealed a main effect of treatment [*F*(4,81) = 6.259; *p* < 0.001] in the L_15_ condition only; the Holm–Sidak test indicated that the groups P and T + P entered less frequently into the open arms, compared to the Nv, V, and T groups. Statistical analysis also detected interactions between the treatment and lactation condition [*F*(4,81) = 3.339, *p* = 0.014], revealing that the group T-L_15_ entered the open arms a higher number of times and the group T + P-L_15_ entered a lower number of times when compared to their respective L_9_ groups.

In relation to the total distance traveled by the animals in the EPM, ANOVA identified a main effect of the lactation condition [*F*(1,80) = 5.445, *p* = 0.022], treatment [*F*(4,80) = 19.010, *p* < 0.001] and an interaction between these two factors [*F*(4,80) = 2.697, *p* = 0.036]. The T-L_9_ group traveled a greater distance compared to the groups Nv, V, P, and T + P of the same lactation condition and the group P-L_9_, a smaller distance compared to Nv, V, and T-L_9_. In the L_15_ condition the groups P and T + P ran a smaller distance compared to the groups Nv, V, and T. The group Nv-L_15_ ran a greater distance when compared to the group Nv-L_9_.

Analysis of variance (ANOVA) identified a main effect of treatment for the ***total immobility*** of the animals [*F*(4,81) = 8.137; *p* < 0.001] and the Holm–Sidak test revealed that the P group had the longest immobility compared to the Nv, V, and T groups in both lactation conditions.

Regarding the number of fecal boluses expelled by each animal during the behavioral test, there was a main effect of treatment [*F*(4.81) = 6.498, *p* < 0.001] and the Holm–Sidak test revealed that the P-L_9_ group expelled a higher number of fecal boluses compared to the corresponding Nv, V, and T groups. In the L_15_ condition, the P group expelled a higher number of fecal boluses compared with the corresponding T and T + P groups.

### CSD Parameters

#### CSD-Induced ECoG Potentiation

**FIGURE 4:**
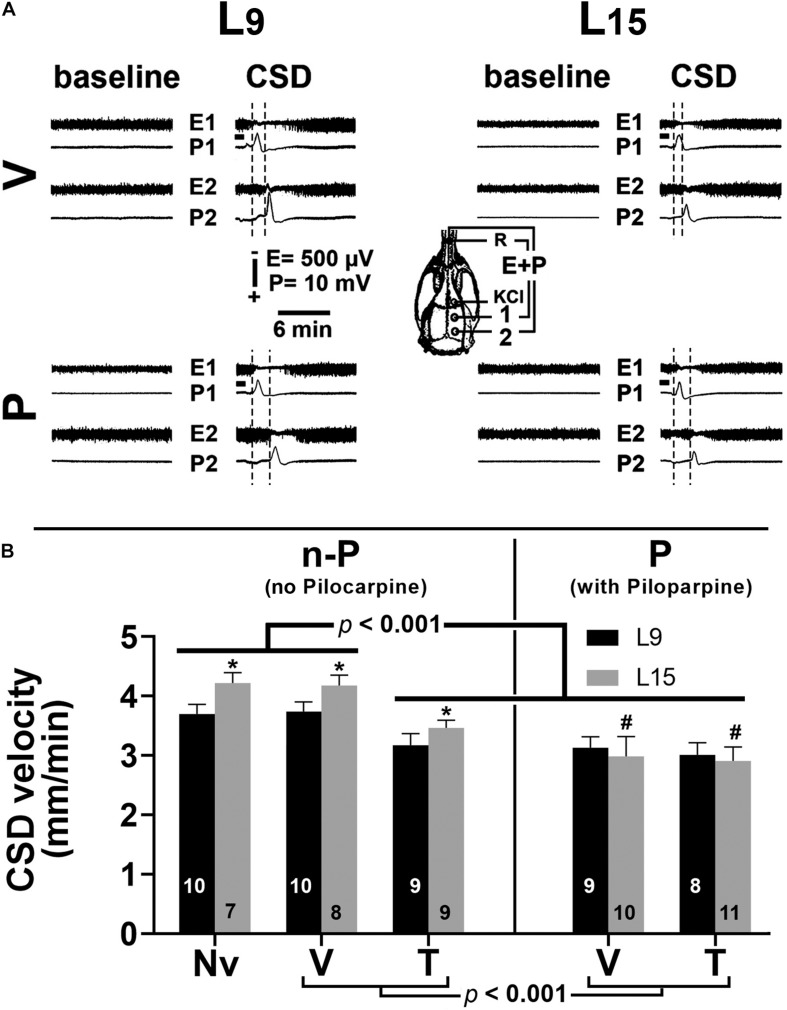
**(A)** Electrocorticogram (E) and slow potential change (P) on two points of the surface of the right hemisphere before (baseline period) and after the passage of cortical spreading depression (CSD period) in four 60–65-day-old male rats previously suckled in litters with 9 and 15 pups (respectively, L_9_ and L_15_ condition). Traces are from two vehicle and two pilocarpine-treated rats in the L_9_ (left) and L_15_ nutritional condition (right). Group description is as in Figure 2. The skull diagram shows the recording positions 1 and 2, from which the traces marked with the same numbers were obtained. The position of the common reference electrode (R) on the nasal bones and the application point of the CSD-eliciting stimulus (KCl) are also shown. CSD was elicited in the frontal cortex by chemical stimulation (a 1- to 2-mm diameter cotton ball soaked with 2% KCl) applied for 1 min on the intact dura mater, as indicated by the horizontal bars. ECoG amplitude in the CSD period is higher (potentiation), in comparison with the baseline amplitude for the same animal. **(B)** Cortical spreading depression velocity (mm/min) of 60–65-day-old male rats previously suckled in litters with 9 and 15 pups (respectively, L_9_ and L_15_ condition). Administration of vehicle, taurine and pilocarpine occurred from postnatal day 35 to 55. Data are mean ± standard deviation. ^∗^*p* < 0.001 compared with the corresponding L_9_ condition. ^#^*p* < 0.001 compared to groups Nv (naïve), V (vehicle), and T (taurine) of the same lactation condition (ANOVA plus Holm–Sidak test). The number in each bar indicates the sample size.

Examples of ECoG amplitude at recording points 1 (E1) and 2 (E2), before and after CSD, are shown in Figure 4A. In all groups, the paired *t*-test showed that for each animal the ECoG amplitude in the CSD period was significantly (*p* < 0.005) higher than in the baseline period (potentiation). This effect was observed for both recording points E1 and E2. The data on CSD-related ECoG potentiation are presented in Table 1.

**TABLE 1:** Amplitude of ECoG, at the recording points 1 and 2, before (baseline period) and during the period of cortical spreading depression elicitation (CSD period), in rats previously suckled in litters with 9 and 15 pups (respectively, condition L_9_ and L_15_).

**Group**	**ECoG 1**		**ECoG 2**	
	**Baseline**	**CSD**	**Baseline**	**CSD**
L_9_				
Naïve	1.01 ± 0.01(10)	1.63 ± 0.25^∗^	1.04 ± 0.04(10)	1.28 ± 0.21^∗^
Vehicle	1.04 ± 0.04(10)	1.67 ± 0.22^∗^	1.04 ± 0.04(10)	1.33 ± 0.14^∗^
Taurine	1.00 ± 0.00(9)	1.40 ± 0.11^∗^#	1.02 ± 0.04(9)	1.32 ± 0.13^∗^
Pilocarpine	1.01 ± 0.03(9)	1.30 ± 0.08^∗^#	1.02 ± 0.03(9)	1.22 ± 0.13^∗^
Taurine + pilocarpine	1.02 ± 0.04(8)	1.43 ± 0.16^∗^#	1.05 ± 0.08(8)	1.35 ± 0.09^∗^
L_15_				
Naïve	1.02 ± 0.04(7)	1.43 ± 0.22^∗^ +	1.03 ± 0.05(7)	1.25 ± 0.15^∗^
Vehicle	1.05 ± 0.05(8)	1.46 ± 0.11^∗^ +	1.01 ± 0.02(8)	1.25 ± 0.14^∗^
Taurine	1.08 ± 0.10(9)	1.47 ± 0.25^∗^	1.04 ± 0.05(9)	1.24 ± 0.09^∗^
Pilocarpine	1.03 ± 0.10(8)	1.21 ± 0.12^∗^§	1.03 ± 0.06(8)	1.16 ± 0.11^∗^
Taurine + pilocarpine	1.03 ± 0.05(11)	1.39 ± 0.10^∗^	1.03 ± 0.06(11)	1.27 ± 0.09^∗^

*The groups received no treatment (naïve; Nv), or vehicle (saline; V), or taurine (T), or pilocarpine (P), or taurine + pilocarpine (T + P). These treatments occurred from PND35 to PND55, and a 6-h long ECoG recording session occurred at PND60-65. In the initial 2 h of the recording session, no CSD was elicited (baseline period). This was followed by 4 h recording, during which CSD was evoked at 30 min intervals (CSD period). From each hour of recording, a 10-min ECoG sample was analyzed by an algorithm implemented in MATLAB^TM^ software, which calculated the average ECoG amplitude. Data are expressed as the mean ± SD and presented as relative units (values of normalized amplitudes with respect to the lowest value, which was considered equal to 1). ^∗^*p* < 0.005 compared to the baseline amplitude in the same lactation condition (paired *t*-test). ^#^*p* < 0.001 compared to the naïve and vehicle groups in the same lactation condition. ^§^*p* < 0.001 compared to the other four groups in the same lactation condition. +*p* < 0.001 compared to the corresponding L_9_ condition (ANOVA followed by the Holm–Sidak test).*

#### CSD Velocity of Propagation

***In the L***_9_ ***animals***, CSD velocities (mean ± SD in mm/min) in the Nv, V, T, P, and T + P groups were respectively 3.71 ± 0.12, 3.73 ± 0.12, 3.15 ± 0.17, 3.12 ± 0.15, and 3.02 ± 0.20. ***In the L***_15_ ***animals***, the CSD velocities for the Nv, V, T, P, and T + P groups were respectively 4.20 ± 0.16, 4.17 ± 0.15, 3.40 ± 0.11, 3.04 ± 0.19, and 2.94 ± 0.22. ANOVA indicated a main effect of the lactation condition [*F*(1,81) = 34.876; *p* < 0.001], and *post hoc* (Holm–Sidak) test comparisons showed that the velocities were higher in the L_15_ groups compared to the L_9_ for the Nv, V, and T groups. ANOVA also detected a main effect of treatment [*F*(4,81) = 150.675; *p* < 0.001], and *post hoc* testing revealed that T, P, and T + P treatment significantly lowered the CSD propagation velocity compared with the corresponding Nv and V controls. The ANOVA revealed interactions between nutritional status and treatment, in which the L_15_ P and T + P groups had lower CSD propagation velocities compared to the Nv, V, and T groups [*F*(4,81) = 12.739, *p* < 0.001], and no difference was observed between the L_9_ and L_15_ animals treated with P and T + P. Data on the CSD propagation velocity are given in Figure 4B.

#### Amplitude and Duration of the CSD Negative Slow Potential Change

Table 2 shows data on the amplitude and duration of the negative slow potential change, which is the hallmark of CSD. ANOVA indicated a main effect of the lactation condition ***on the CSD amplitude*** [*F*(1,81) = 11.408; *p* < 0.001], and a *post hoc* (Holm–Sidak) test comparison showed that the amplitudes were higher in the naïve, vehicle, and taurine L_15_ groups compared to the corresponding L_9_ groups. The factor treatment also affected the amplitude [*F*(4,81) = 3.274; *p* = 0.015], and a *post hoc* test showed that the amplitude was lower in the pilocarpine-treated L_15_, but not in the L_9_ group, compared with the corresponding naïve, vehicle, and taurine of the same lactation condition. ANOVA also confirmed an interaction between both factors [*F*(4,81) = 2.934; *p* = 0.026].

**TABLE 2:** Amplitude and duration of the negative slow potential change of CSD in male rats previously suckled in litters with 9 and 15 pups (respectively, L_9_ and L_15_ condition).

**Group**	**Amplitude (mV)**	**Duration (s)**
L_9_		
Naïve	8.5 ± 1.6(10)	69.2 ± 2.6(10)
Vehicle	8.6 ± 1.0(10)	69.2 ± 2.6(10)
Taurine	8.6 ± 1.2(9)	69.3 ± 2.2(9)
Pilocarpine	8.4 ± 1.1(9)	70.9 ± 2.7(9)
Taurine + pilocarpine	9.3 ± 1.5(8)	70.3 ± 1.9(9)
L_15_		
Naïve	10.6 ± 3.1(7)^∗^	64.7 ± 0.6(7)^∗^
Vehicle	10.8 ± 1.8(8)^∗^	65.1 ± 0.8(11)^∗^
Taurine	10.7 ± 1.6(9)^∗^	65.1 ± 0.5(9)^∗^
Pilocarpine	7.5 ± 1.9(10) +	67.9 ± 1.0(10)^∗^#
Taurine + pilocarpine	9.6 ± 2.2(11)	65.2 ± 1.3(11)^∗^

*Naïve = no treatment; Vehicle = scopolamine methyl nitrate 1 mg/kg/day + saline; Taurine = scopolamine methyl nitrate 1 + 300 mg/kg/day of taurine; Pilocarpine = scopolamine methyl nitrate 1 + 45 mg/kg/day of pilocarpine; Taurine + pilocarpine = scopolamine methyl nitrate 1 + 300 mg/kg/day of taurine + 45 mg/kg/day of pilocarpine. All drugs were dissolved in 0.9% saline. Data are expressed as the mean ± standard deviation, with the number of animals in parentheses. ^∗^*p* < 0.001 compared with the corresponding L_9_ condition. ^#^*p* = 0.015 compared with naïve, vehicle, and taurine groups in the same suckling condition. +*p* < 0.001 compared with all other groups in the same suckling condition (ANOVA plus Holm–Sidak test).*

Analysis of ***CSD duration*** indicated a main effect of the lactation condition [*F*(1,81) = 115.827; *p* < 0.001] and treatment [*F*(4,81) = 5.291; *p* < 0.001]. The Holm–Sidak test indicated a shorter duration in the L_15_ groups compared with the corresponding L_9_ groups and a longer duration in the L_15_ pilocarpine-treated animals compared with the corresponding naïve, vehicle, taurine, and taurine + pilocarpine of the same lactation condition.

#### MDA Levels in the Cortex and Hippocampus

**FIGURE 5:**
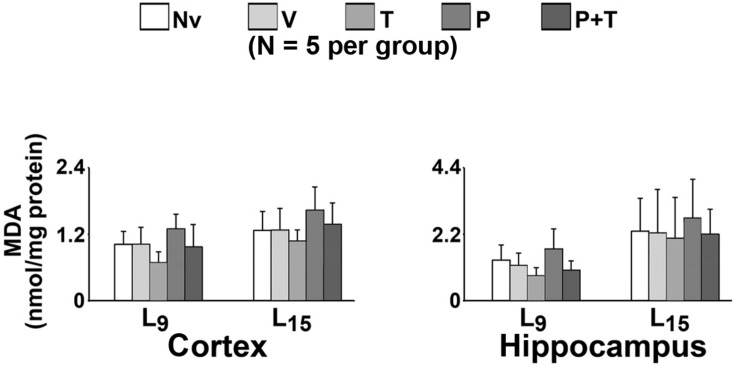
Malondialdehyde (MDA) levels (nmol/mg protein) of 60–65-day-old male rats previously suckled in litters with 9 and 15 pups (respectively, L_9_ and L_15_ condition). Group description as in Figure 2. Data are mean ± standard deviation of 5 rats per group. Measurements were performed in triplicate. No significant differences were observed.

Measurements of MDA levels in the cerebral cortex and hippocampus are shown in Figure 5. ANOVA revealed no significant differences. MDA levels ***in the cortex*** in the L_9_ animals (mean ± SD in nmol/mg of protein) in the naïve, vehicle, taurine, pilocarpine, and taurine + pilocarpine groups were respectively 1.02 ± 0.23, 1.02 ± 0.30, 0.68 ± 0.20, 1.30 ± 0.26, and 0.97 ± 0.42. In the L_15_ animals, the MDA levels were respectively 1.27 ± 0.34, 1.27 ± 0.39, 1.07 ± 0.20, 1.63 ± 0.42, and 1.36 ± 0.39.

Malondialdehyde levels ***in the hippocampus*** in the L_9_ animals (mean ± SD in nmol/mg of protein) in the naïve, vehicle, taurine, pilocarpine, and taurine + pilocarpine groups were respectively 1.34 ± 0.50, 1.16 ± 0.41, 0.84 ± 0.25, 1.72 ± 0.66 and 1.00 ± 0.31. In the L_15_ animals, the MDA levels were respectively 2.30 ± 1.08, 2.24 ± 1.44, 2.06 ± 1.36, 2.74 ± 1.26, and 2.21 ± 0.80.

#### Immunohistochemistry for Microglia and Astrocytes

**FIGURE 6:**
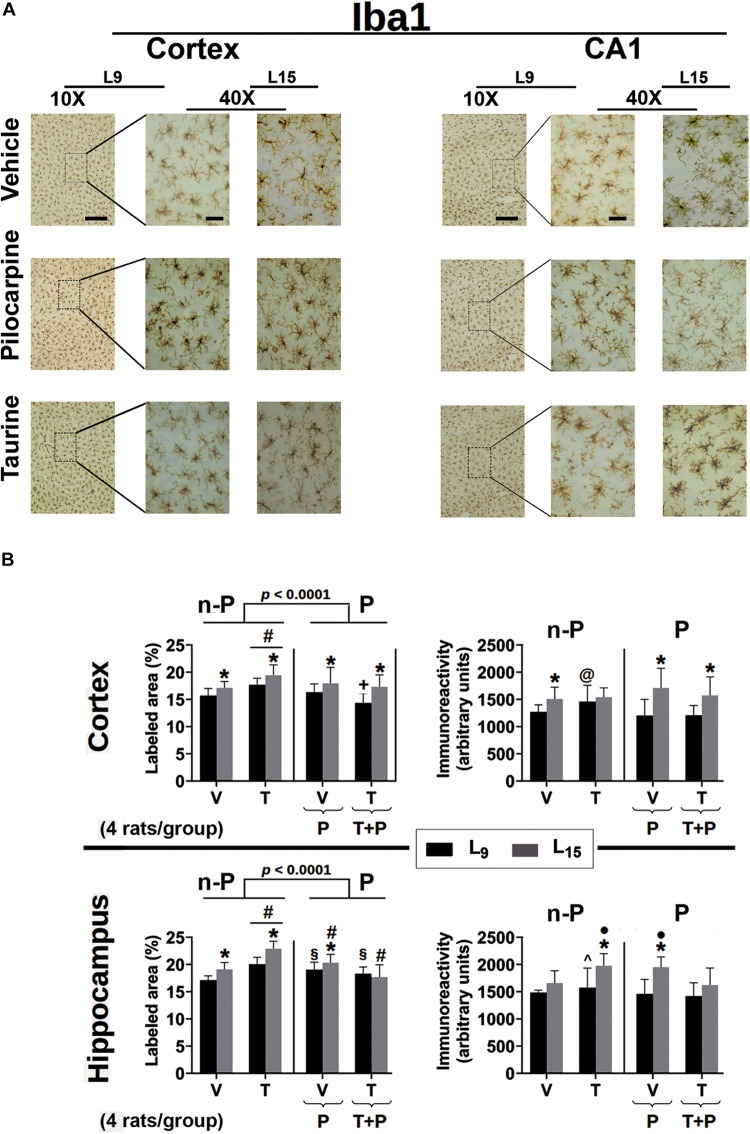
**(A)** Photomicrographs of immunolabeled Iba1-positive cells in the left cortex and hippocampus of 60–65-day-old male rats previously suckled in litters with 9 and 15 pups (respectively, L_9_ and L_15_ condition). Group description is as in Figure 2. Scale bars = 50 and 10 μm for the low (10×) and high magnification pictures (40×), respectively. **(B)** Percent labeled area (left panels) and immunoreactivity as arbitrary units (right panels) of immunolabeled Iba1-positive cells in the cortex (upper panels) and hippocampus (lower panels) of 60–65-day-old male rats previously suckled in litters with 9 and 15 pups (respectively, L_9_ and L_15_ condition). Group description is as in Figure 2. n-P = no pilocarpine; P = with pilocarpine. Data are mean ± standard deviation of four rats per group. ^∗^*p* < 0.001 compared to the corresponding L_9_ condition. ^§^ *p* < 0.001 compared to groups V in the same lactation condition. ^#^*p* < 0.001 compared to the other three groups in the same lactation condition. +*p* < 0.001 compared to groups V and P of the same lactation condition. ^∙^*p* < 0.001 compared to groups V and T + P of the same lactation condition. @*p* < 0.001 compared to groups P and T + P of the same lactation condition. ^∧^*p* < 0.001 compared to group Nv of the same lactation condition (ANOVA followed by the Holm–Sidak test).

The effect of administration of taurine and/or pilocarpine on the percentage of labeled area and immunoreactivity of astrocyte and microglia cells in the parietal cortex and CA1 hippocampus is shown in the Figure 6 for Iba1, and 7 for GFAP. All data are described as the mean ± standard deviation from four animals per group.

#### Iba-1 Immunohistochemistry

Data from Iba1 immunohistochemistry are presented in Figure 6. In the ***cerebral cortex***, ANOVA showed in all groups a main effect of the lactation condition on the ***percentage of Iba1-labeled area*** [*F*(1,371) = 5.074; *p* < 0.001] and *post hoc* (Holm–Sidak) test comparisons showed that the animals of the L_15_ condition had a greater percentage of labeled area when compared to the corresponding L_9_ groups. ANOVA also detected a main effect of treatment [*F*(4,371) = 23.681; *p* < 0.001], and *post hoc* testing revealed in the two lactation conditions that taurine treatment significantly increased the percentage of labeled area, compared to the other four treatment groups. On the other hand, in the L_9_ condition, the taurine + pilocarpine treated group presented with a lower percentage of the labeled area when compared with the naïve, vehicle, and pilocarpine-treated groups. Regarding ***the Iba1 immunoreactivity in the cerebral cortex***, ANOVA indicated a main effect of the lactation condition [*F*(1,355) = 59.454; *p* < 0.001] and *post hoc* testing comparisons showed that the animals of the L_15_ condition had greater immunoreactivity when compared to the corresponding L_9_ condition, ***except for the taurine-treated groups***. ANOVA also revealed interactions between nutritional status and treatment, in which the pilocarpine and taurine + pilocarpine groups ***in the L***_15_, ***but not in the L***_9_ ***condition*** had higher immunoreactivity compared to the other groups [*F*(4,355) = 5.954; *p* < 0.001].

***In the hippocampus***, ANOVA revealed a main effect of the lactation condition on the percentage of the Iba-1 labeled area [*F*(1,376) = 64.536; *p* < 0.001]; the *post hoc* (Holm–Sidak) test comparisons showed that the groups naïve, vehicle, taurine, and pilocarpine in the L_15_ condition had a greater percentage of labeled area when compared to the corresponding L_9_ groups. ANOVA revealed a main effect of treatment [*F*(4,376) = 65.946; *p* < 0.001], and *post hoc* testing detected that in the two lactation conditions taurine treatment significantly increased the percentage of Iba1 labeled area compared to the other four groups.

Furthermore, in the L_9_ condition, pilocarpine and taurine + pilocarpine treatments resulted in a higher percentage of labeled area when compared with the L_9_ naïve and vehicle. In the L_15_ condition, the pilocarpine group displayed a percentage of labeled area that was greater than in the naïve, vehicle and taurine + pilocarpine groups, but was lower than in the taurine group; the L_15_ taurine + pilocarpine group had the lowest percentage of Iba1-labeled area. ANOVA also revealed interactions between nutritional status and treatment [*F*(4,376) = 11.742; *p* < 0.001], in which the L_15_ taurine group had a higher percentage of Iba1 labeled cortical area compared to the other four groups.

Regarding the ***Iba-1 immunoreactivity in the hippocampus***, ANOVA indicated a main effect of the lactation condition [*F*(1,350) = 59.217; *p* < 0.001] and *post hoc* testing comparisons showed that the animals of the groups naïve, taurine, and pilocarpine in the L_15_ condition had greater immunoreactivity when compared to the corresponding L_9_ groups. ANOVA revealed a main effect of taurine treatment [*F*(4,350) = 14.626; *p* < 0.001], and *post hoc* testing showed in the L_9_ condition a significantly greater immunoreactivity of the taurine-treated animals when compared to the corresponding naïve group. ***Among the L***_15_ ***animals***, the taurine- and pilocarpine-treated groups had higher Iba1 immunoreactivity compared with the naïve, vehicle, and taurine + pilocarpine groups. ANOVA also identified interactions between nutritional status and treatment, in which the L_15_ taurine and pilocarpine groups had higher immunoreactivity compared to the other L_15_ groups [*F*(4,350) = 4.545; *p* < 0.001].

#### GFAP Immunohistochemistry

**FIGURE 7:**
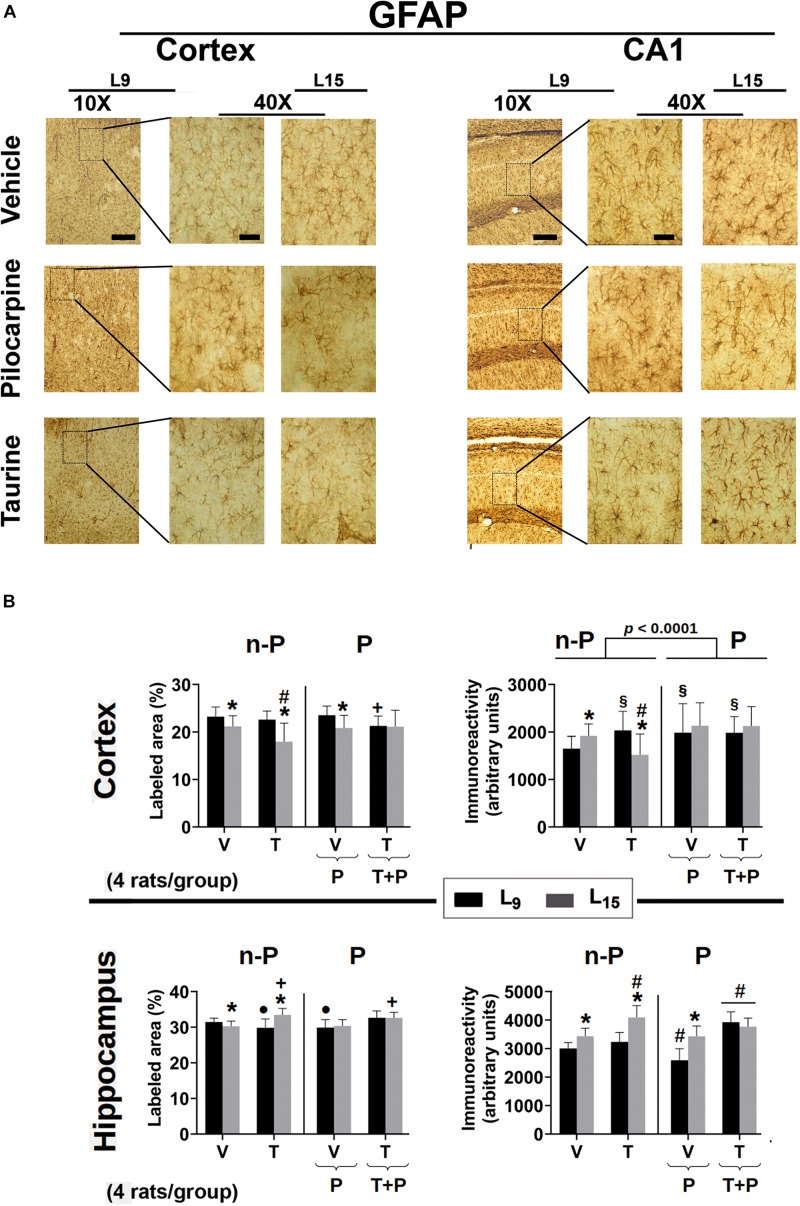
**(A)** Photomicrographs of immunolabeled GFAP-positive cells in the left cortex and hippocampus of 60–65-day-old male rats previously suckled in litters with 9 and 15 pups (respectively, L_9_ and L_15_ condition). Group description is as in Figure 2. Scale bars = 50 and 10 μm for the low (10×) and high magnification pictures (40×), respectively. **(B)** Percent labeled area (left panels) and immunoreactivity as arbitrary units (right panels) of immunolabeled GFAP-positive cells in the cortex (upper panels) and hippocampus (lower panels) of 60–65-day-old male rats previously suckled in litters with 9 and 15 pups (respectively, L_9_ and L_15_ condition). Group description is as in Figure 2. n-P = no pilocarpine; P = with pilocarpine. Data are mean ± standard deviation of four rats per group. ^∗^*p* < 0.001 compared to the corresponding L_9_ condition. ^§^ *p* < 0.001 in comparison to group V in the same lactation condition. ^#^*p* < 0.001 compared to the other three groups in the same lactation condition. ^+^*p* < 0.001 compared to groups V and P of the same lactation condition. ^∙^*p* < 0.001 compared to groups V and T + P of the same lactation condition (ANOVA followed by the Holm–Sidak test).

Data on GFAP immunohistochemistry are shown in Figure 7. ANOVA showed a main effect of the lactation condition on the ***percentage of GFAP-labeled areas in the cerebral cortex*** [*F*(1,434) = 63.276; *p* < 0.001] and *post hoc* (Holm–Sidak) test comparisons showed that the naïve, vehicle, and pilocarpine-treated groups of the L_15_ condition had a lower percentage of labeled area when compared to the corresponding L_9_ groups. ANOVA also indicated a main effect of treatment [*F*(4,434) = 6.988; *p* < 0.001], and *post hoc* testing revealed that, in the L_9_ condition, taurine + pilocarpine treatment significantly lowered the percentage of labeled area compared to the naïve, vehicle and pilocarpine groups, and in the L_15_ condition, taurine treatment resulted in a lower percentage of labeled area when compared with the other four groups in the L_15_ condition. ANOVA also revealed interactions between nutritional status and treatment [*F*(4,434) = 5.744; *p* < 0.001], in which the L_15_ taurine group had a lower percentage of labeled area in comparison with all other L_15_ groups.

In relation to GFAP ***immunoreactivity quantification in the cerebral cortex***, in the L_9_ condition ANOVA revealed a main effect of treatment [*F*(4,434) = 6.147; *p* < 0.001], and *post hoc* testing showed greater immunoreactivity in the groups treated with taurine, pilocarpine, and taurine + pilocarpine when compared to the naïve and vehicle-treated controls. Among ***the L***_15_ ***groups***, taurine animals presented with lower immunoreactivity compared to the other four groups. ANOVA also demonstrated interactions between nutritional status and treatment, in which the L_15_ taurine group had lower immunoreactivity compared to the corresponding naïve, vehicle groups, and with the taurine L_9_ group [*F*(4,434) = 10.551; *p* < 0.001].

Regarding ***GFAP-labeled cells in the hippocampus***, in the L_9_ condition ANOVA showed a main effect of treatment [*F*(4,401) = 14.819; *p* < 0.001], and *post hoc* testing revealed that taurine and pilocarpine treatments significantly lowered the percentage of labeled area compared to the naïve, vehicle, and taurine + pilocarpine groups. ***In the L***_15_ ***condition***, taurine and taurine + pilocarpine treatments resulted in a higher percentage of the labeled area when compared with the naïve, vehicle, and pilocarpine groups. ANOVA also revealed interactions between nutritional status and treatment [*F*(4,401) = 15.918; *p* < 0.001], in which the taurine treatment resulted in a greater percentage of labeled area in the L_15_ but not in the L_9_ condition. Regarding the ***GFAP immunoreactivity in the hippocampus***, ANOVA detected a main effect of the lactation condition [*F*(1,359) = 107.184; *p* < 0.001], and the *post hoc* testing comparisons showed that the groups naïve, vehicle, taurine, and pilocarpine in the L15 condition had greater immunoreactivity when compared to the corresponding L9 groups. The ANOVA detected a main effect of treatment [*F*(4,359) = 53.366; *p* < 0.001], and *post hoc* testing showed a significantly lower immunoreactivity in the L_9_ pilocarpine group compared to the other L_9_ groups. *In the L_15_ condition*, taurine animals presented with greater immunoreactivity compared to the other four L_15_ groups. In both lactation conditions, taurine + pilocarpine treatment resulted in greater immunoreactivity compared to the other nutrition-matched treatment groups. ANOVA also detected interactions between nutritional status and treatment, in which the L_15_ taurine group had greater immunoreactivity compared to the corresponding L_9_ group [*F*(4,359) = 21.074; *p* < 0.001].

## Discussion

The present study extends the previous observations of our group on the effects of taurine (Francisco and Guedes, 2015) and pilocarpine (Mendes-da-Silva et al., 2018) in rats of a younger age (PND7-27) than our rats (PND35-55). Analyzing such effects at another age might be of importance, regarding the time course of drug actions (Guedes and Abadie-Guedes, 2019). Treatment with taurine and pilocarpine resulted in anxiolytic-like and anxiogenic behavior, respectively. In addition, chronic pilocarpine, which was administered on a subconvulsing basis, reduced body weight and glycemia; both taurine and pilocarpine treatment decelerated CSD propagation. Furthermore, these data extend our knowledge about the actions of taurine and pilocarpine on electrophysiological responses and glial cell reactions, as demonstrated by the CSD-related potentiation of ECoG amplitude and microglial and astrocyte immunoreactivity. The previously described *in vivo* CSD-induced ECoG potentiation (Lopes-de-Morais et al., 2014; Mendes-da-Silva et al., 2018) has been confirmed in this paper, and its modulation by pilocarpine and taurine has been described (Table 1). Data on anxiety-like behavior, MDA measurements and brain immunohistochemical effects that were produced by taurine and/or pilocarpine treatment represent novel evidence of the action of these compounds on 35–55-day-old rats, whereas data on weight gain, glycemia, and CSD confirm the results of a previous study (Francisco and Guedes, 2018). The stress that is associated with drug administration cannot be the cause of the reported alterations because – as first mentioned in the results section – the groups that received gavage and intraperitoneal injection of vehicle (saline and scopolamine) presented values similar to the naïve controls that received no gavage or intraperitoneal injection. The results collectively emphasize the effectiveness of taurine and pilocarpine treatment in modulating behavioral and electrophysiological parameters of the brain, as well as glial cell reactivity.

The ponderal reduction of body and brain weights in the animals under the L_15_ lactation condition (Figure 2) are consistent with our previous data (Francisco and Guedes, 2015, 2018), confirming the effectiveness of increasing litter size in producing malnutrition during the suckling period (Rocha-de-Melo et al., 2006). In early life, the brain is more vulnerable to environmental challenges, such as unfavorable lactation conditions (Morgane et al., 1978; Rocha-de-Melo et al., 2006). As previously pointed out, the evidence indicates that a body weight reduction implies weight diminution in the brain and other organs, which is usually associated with alterations in the organs’ function (Morgane et al., 2002). In animal models, nutritional deficiency early in life can permanently (or at least in a long-lasting manner) reduce the number of synapses, diminish myelin production, decrease the size and/or number of brain cells, and alter neurotransmitter systems (Morgane et al., 2002). As previously suggested (Francisco and Guedes, 2015), alterations in these processes may interfere with behavioral, electrophysiological and immunohistochemical parameters such as those presently investigated.

Experimental alteration of the blood glucose levels has been shown to correlate inversely with the brain’s ability to produce and propagate CSD (Ximenes-da-Silva and Guedes, 1991; Hoffmann et al., 2013), and the present data are in line with the literature. Our interpretation is that an adequate glucose supply is crucial in providing the energy necessary for the glial mechanisms that counteract CSD propagation, as CSD is an energy-demanding phenomenon (Back et al., 1994; Dohmen et al., 2008).

Elevated plus-maze test is one of the most commonly used behavioral tests to evaluate aspects of anxiety-like reactions in rodents (Ramos, 2008). Our findings indicated that treatment with pilocarpine and taurine were associated with anxiogenic and anxiolytic behavior, respectively, as judged by the frequency and duration of the animals’ visiting the open arms of the EPM (Figure 3). Our EPM data on taurine and pilocarpine administration are consistent with previous reports that showed an anxiolytic effect of taurine administration on animals tested in the EPM (Kong et al., 2006; Murakami and Furuse, 2010; Francisco and Guedes, 2015), and pilocarpine-associated anxiogenic behavior in rats tested in the EPM (Castelhano et al., 2015) and open field apparatus (Francisco and Guedes, 2018). Therefore, we think that it is possible that taurine’s action in the brain counteracts anxiety, as suggested by others (Murakami and Furuse, 2010; Yu et al., 2015). By the same logic, chronic pilocarpine at a subconvulsing dose seems to be anxiogenic (present data). Considering that the pilocarpine dose in this study (45 mg/kg/day) was very low (corresponding to 12–15% of the convulsing dose), we suggest that pilocarpine-induced anxiogenesis does not necessarily require the generation of convulsive episodes, as previously indicated (Duarte et al., 2014).

Nutrient intake in insufficient quantity and/or quality in early life can negatively modify structural, biochemical, and electrophysiological parameters of the brain and these effects may last into adult life (Morgane et al., 1978; Guedes, 2011). In rats, it is well-established that early-in-life malnutrition accelerates CSD propagation, both when malnutrition was induced by maternal diet manipulation (Mendes-da-Silva et al., 2014, 2018) and in an unfavorable lactation condition (Lima et al., 2017; Francisco and Guedes, 2018; present study). The mechanisms by which malnutrition facilitates CSD propagation are still a matter of debate. While a larger volume of brain extracellular space hinders CSD elicitation and propagation (Mazel et al., 2002), malnutrition in early life may increase cell-packing density and reduce the extracellular space in the brain (Morgane et al., 1978, 2002), which may facilitate CSD propagation (Lima et al., 2017). Furthermore, malnutrition reduces brain myelination (Morgane et al., 1978). It is important to note that CSD propagation velocity is increased in the myelin-deficient cortex and decreased in hypermyelinated knock-in animals (Merkler et al., 2009). In addition, early malnutrition may induce impairment of glial function (Morgane et al., 1978), and this condition has been shown to accelerate CSD (Largo et al., 1997). Malnutrition can also enhance the brain levels of the enzyme glutamic acid decarboxylase (Díaz-Cintra et al., 2007) and reduce brain glutamate uptake (Feoli et al., 2006), constituting a scenario that is favorable to CSD elicitation and propagation (Marrannes et al., 1988).

Regarding the redox balance in the brain, previous data suggested that CSD can induce oxidative stress (Viggiano et al., 2011; Shatillo et al., 2013). In the other direction, the accumulation of reactive oxygen species in the nervous system can trigger CSD (Netto and Martins-Ferreira, 1989; El-Bachá et al., 1998; Malkov et al., 2014). Interestingly, various antioxidant agents have been shown to counteract CSD (Abadie-Guedes et al., 2012, 2016; Lopes-de-Morais et al., 2014). As referred above (see introduction), taurine exhibits antioxidant properties and plays a neuroprotective role in epilepsy (Junyent et al., 2011). Because taurine is able to cross the blood–brain barrier, its level in the brain is increased when taurine is administered systemically (Menzie et al., 2013). Taken together, these pieces of evidence could explain the decelerating action of taurine on CSD, as taurine is considered a molecule with antioxidant properties (El-Maraghi et al., 2018; Jafri et al., 2019; Ommati et al., 2019). When administered to pregnant rat dams, taurine is capable of attenuating the impact of maternal food restriction on the progeny (Wang et al., 2017). Our data reinforce a recent suggestion that, in an excitability imbalance condition, taurine plays an inhibitory and neuroprotective role (Hrnčić et al., 2018). As a molecular structure that is very similar to the neurotransmitter GABA, taurine affects the opening of chloride channels, preferably by interactions with GABA_*A*_ receptors and with lower affinity to the glycine and GABA_*B*_ receptors in the adult brain (Oja and Saransaari, 2013; Hrnčić et al., 2018). Interestingly, extracellular chloride imbalance has been shown to affect CSD propagation *in vitro* in the isolated retina (Martins-Ferreira et al., 1974) and *in vivo* in the rabbit cortex (Guedes and Do Carmo, 1980).

Pilocarpine-induced seizures are correlated with elevated production of reactive oxygen species and by cerebral amino acid level changes, with increased glutamate content and decreased taurine levels in the rat hippocampus (Santos et al., 2011). On the other hand, chronic treatment with a subconvulsive dose of pilocarpine (45 mg/kg/day) did not increase MDA levels in the brain tissue (Mendes-da-Silva et al., 2018), suggesting that under those conditions oxidative stress does not increase, and the present data reinforces this suggestion. Taken together, the findings support the hypothesis that, after pilocarpine administration, brain levels of MDA increase only if pilocarpine induces convulsive seizures (Hamed and Abdellah, 2004; Freitas et al., 2010; Carmona-Aparicio et al., 2016; McElroy et al., 2017).

Microglial cells have the ability to react in response to CNS demands, such as those in epileptic disorders (Eyo et al., 2017) and nutritional imbalance (Rideau Batista Novais et al., 2016). In this study, unfavorable suckling (in large litters) increased microglia activation in the cortex and hippocampus, in addition to the behavioral (anxiety) and electrophysiological (CSD) effects, corroborating the findings of previous studies (Viana et al., 2013; Lima et al., 2017). Furthermore, in line with taurine’s effects on traumatic brain injury (Su et al., 2014), our taurine treatment decreased glial immunoreactivity in the cortex and hippocampus. Interestingly, both the cortex and hippocampus are pathologically affected regions in human temporal lobe epilepsy (Gonçalves Pereira et al., 2005; Das et al., 2009). In the pathogenesis of this disease, astrocyte and microglial cell activation is an important link (Clasadonte et al., 2016; Zhao et al., 2018).

Microglia cells may be activated, not merely by delayed neuronal degeneration, but by neuronal hyperactivity that precedes neuronal demise (Eyo et al., 2017). These cells are the resident immune cells of the CNS. They continuously survey the microenvironment with their highly dynamic processes, thus being able to become activated in response to tissue requirements associated with deleterious conditions, such as those involved in brain inflammatory and behavioral responses (Viana et al., 2013). It is interesting to note that Iba1 immunolabeling also labels macrophages, which may infiltrate the CNS; therefore, it is not a specific marker for microglia, and macrophages may become involved in inflammatory responses in the brain, as well as in behavioral responses (Wohleb et al., 2015).

Similar to microglia, astrocyte activation occurs following nutrition imbalance and seizures, and plays an important role in epileptogenesis. A recent study showed that early in life nutritional imbalance can have a lasting impact on astrocytes. It seems that protein deprivation decreased GFAP expression, whereas other undernutrition models (e.g., large litter size) increased GFAP expression (Abbink et al., 2019). In the temporal lobe epilepsy, hippocampal astrocytes undergo dramatic morphological and molecular changes, similar to the glial morphological changes in the hippocampus seen in the pilocarpine-induced epilepsy model (Shapiro et al., 2008). These hippocampal changes occur during the early latent stage of the pilocarpine model. Regarding the cortical astrocytes however, it has been suggested that molecular changes could be a consequence of the repetitive occurrence of seizures (Clasadonte et al., 2016).

In our study, in the L_15_ condition taurine increased GFAP immunoreactivity compared to the L_15_ control groups. It appears that this effect was not harmful to the animals, since in this group the behavioral and electrophysiological parameters were not impaired. Increased immunoreactivity for GFAP has been observed in healthy animals that were subjected to an enriched environment and treated with taurine, suggesting an interaction between these two factors (Rahmeier et al., 2016). Behaviorally, taurine supplementation in rats can improve stress-induced cognitive dysfunction (Jia et al., 2016). Surprisingly, we found lower immunoreactivity for GFAP in the hippocampus of L_9_ pilocarpine-treated rats. Borges et al. (2006) reported transient loss of astrocytes in mice dentate hilus early after pilocarpine-induced status epilepticus. In rats, they observed about 50% reduction in hilar neurons but no change in astrocyte number (Borges et al., 2006). Another study reported that a sub-threshold dose of pilocarpine (15 mg/kg), following a lithium chloride administration (127 mg/kg), increases GFAP immunoreactivity in the hippocampus (Sun et al., 2016). In view of the controversial reports, we think that further research is necessary for the complete understanding of the effect of non-convulsing dose of pilocarpine on astrocytes.

## Conclusion

The results of this study suggest a brain effect of taurine in pilocarpine-treated rats, which is expressed in body weight, glycemia, behavioral (anxiety), electrophysiological parameters related with CSD and glial alterations. For some of these parameters, the data suggest taurine/pilocarpine/malnutrition interactions, whose mechanisms deserve further exploration.

## Data Availability

All datasets generated for this study are included in the manuscript and/or the supplementary files.

## Ethics Statement

This study was carried out in accordance with the recommendations of the Ethics Committee for Animal Research of the Federal University of Pernambuco, Brazil. The protocol was approved by the same committee (approval protocol no. 23076.015655/2015-99).

## Author Contributions

EF and RG conceived the study, performed the experiments, analyzed the data, and wrote the manuscript. RM conducted the biochemical analysis and helped in manuscript revision. GS and CC conducted the immunohistochemical analysis, and helped in manuscript revision and in preparing some illustrations.

## Conflict of Interest Statement

The authors declare that the research was conducted in the absence of any commercial or financial relationships that could be construed as a potential conflict of interest.
